# Influence of blood group, Glucose-6-phosphate dehydrogenase and Haemoglobin genotype on *Falciparum* malaria in children in Vihiga highland of Western Kenya

**DOI:** 10.1186/s12879-020-05216-y

**Published:** 2020-07-09

**Authors:** Jafaralli Sande Ahmed, Bernard Guyah, David Sang’, Mark Kilongosi Webale, Nathan Shaviya Mufyongo, Elly Munde, Collins Ouma

**Affiliations:** 1 Department of Biomedical Sciences and Technology, Maseno University, Maragoli, Kenya; 2 Department of Health, County Government of Vihiga, Vihiga, Kenya; 3 School of Health Sciences, Kirinyaga University, Kerugoya, Kenya; 4 Department of Biomedical Sciences and Technology, Masinde Muliro University of Science and Technology, Kakamega, Kenya

**Keywords:** Genetic variants, Malaria, Children, Western Kenya

## Abstract

**Background:**

Genetic diversity of ABO blood, glucose-6-phosphate dehydrogenase (G6PD) deficiency and haemoglobin type and their ability to protect against malaria vary geographically, ethnically and racially. No study has been carried out in populations resident in malaria regions in western Kenya.

**Method:**

A total of 574 malaria cases (severe malaria anaemia, SMA = 137 and non-SMA = 437) seeking treatment at Vihiga County and Referral Hospital in western Kenya, were enrolled and screened for ABO blood group, G6PD deficiency and haemoglobin genotyped in a hospital-based cross-sectional study.

**Result:**

When compared to blood group O, blood groups A, AB and B were not associated with SMA (*P* = 0.380, *P* = 0.183 and *P* = 0.464, respectively). Further regression analysis revealed that the carriage of the intermediate status of G6PD was associated with risk to SMA (OR = 1.52, 95%CI = 1.029–2.266, *P* = 0.035). There was, however, no association between AS and SS with severe malaria anaemia. Co-occurrence of both haemoglobin type and G6PD i.e. the AA/intermediate was associated with risk to SMA (OR = 1.536, 95%CI = 1.007–2.343, *P* = 0.046) while the carriage of the AS/normal G6PD was associated with protection against SMA (OR = 0.337, 95%CI = 0.156–0.915, *P =* 0.031).

**Conclusion:**

Results demonstrate that blood group genotypes do not have influence on malaria disease outcome in this region. Children in Vihiga with blood group O have some protection against malaria. However, the intermediate status of G6PD is associated with risk of SMA. Further, co-inheritance of sickle cell and G6PD status are important predictors of malaria disease outcome. This implies combinatorial gene function in influencing disease outcome.

## Background

Despite the expanded use of proven malaria control strategies, over 216 million malaria cases and 0.5 million malaria related deaths have been reported worldwide [1]. Africa continues to account for about 90% of all malaria cases and deaths worldwide [1]. This high rates of malaria related morbidities is greatly driven by the malaria vaccine escape and drug resistant mutants resulting from host immune and drug pressure [2]. Therefore, many investigations have been conducted to find out whether or not ABO blood group antigens, glucose-6-phosphate dehydrogenase (G6PD) deficiency and hemoglobin genotypes are associated with susceptibility, resistance, or severity of *P. falciparum* malaria [3].

Rosetting is characterized by the binding of *P. falciparum*-infected red blood cells to uninfected red blood cells to form clusters of cells that are thought to contribute to the pathology of falciparum malaria by obstructing blood flow in small blood vessels [4]. Rosetting parasites such as *P. falciparum* form larger, stronger rosettes in non-O (A, B or AB) blood groups than in group O red blood cells [5, 6] suggesting that rosetting phenotypes correlates with severe *falciparum* malaria [7]. On the other hand, it has been reported that rosettes form better depending on the blood cell types, with the blood cell type A and B having higher chances of forming rosettes [5, 6]. Some studies have reported the absent of significant association between ABO blood group and malaria [8] while others have reported high frequency of malaria episodes in blood group A, AB, and B compared with other blood group individuals [9].

Impaired rosette formation due to increased sickling or reduced expression of erythrocyte surface adherence proteins in *P. falciparum*-infected HbAS and HbSS red blood cells contribute to protection against malaria [10, 11]. Cyto-adherance enables parasites to sequester in the vasculature and avoid clearance by the spleen, leading to endothelial activation and associated inflammation in the brain and other organs, important in the progression to severe malaria anaemia [12]. However, the link between haemoglobin AA, AS, and SS type and the incidence of malaria parasitaemia or immunity to malaria is still unclear in Kenya. Although some studies have reported that haemoglobin S genotype plays a significant role in modulating malaria in children, others have not [13, 14].

Increased oxidative stress impairs *P. falciparum* growth as well as accelerating ring stage erythrocyte senescence promoting phagocytic clearance and eryptosis of parasitized G6PD deficient cells [15–17] supporting the protection hypothesis. However, there is conflicting information on the effect of G6PD variant on *falciparum* malaria. Some studies have shown that G6PD normal are more vulnerable to *Plasmodium falciparum* malaria than the G6PD deficiency and heterozygous individuals [18], whereas others have reported an equal vulnerability among the various G6PD types [14].

Protection by AS haemoglobin genotype and G6PD deficiency against *falciparum* malaria is usually thought to act independently [17, 19]. In Mali, hemizygous G6PD (A−) condition in the male while sickle cell trait in female children is associated with protection against severe malaria anaemia [20]. Heterozygous G6PD (A−) interfered with the protective effect of haemoglobin AS in females while no evidence of negative epistasis between sickle trait and G6PD (A−) heterozygosity in males of the same population [20]. To our knowledge, however, no study has reported on the concurrent effect of haemoglobin and G6PD variant on malaria. Taken together, variations in reports in the association between red blood cell, haemoglobin, and G6PD type with malaria disease progression shows the complexity of interaction between parasite and host genetics and immunity factors [21, 22]. Moreover, the acquisition of relative immunity with age greatly confounds the influence of ABO blood group, G6PD and haemoglobin genotype on malaria [22]. Since young children have under-developed immunity against malaria and genetic diversity of G6PD, ABO blood groups and sickle cell trait and their ability to protect against malaria vary by region [23–26], it is important to determine the association between G6PD, ABO blood group and haemoglobin genotype with malaria among children in Kenya. As such, the present study determined effect of blood group, glucose-6-phosphate dehydrogenase and haemoglobin genotypes on *P. falciparum* malaria in children in Vihiga highland of western Kenya.

## Methods

### Study area and design

A cross-sectional study targeting children less than 3 years seeking treatment at Vihiga County Referral hospital, Vihiga, Western Kenya was carried out. Study participants were categorised as severe malaria anaemia (SMA; Hb < 5.0 g/dL, with any parasite density) and non-severe malaria anaemia (Non-SMA; Hb ≥ 5.0 g/dL, with any parasite density) There has been a marked increase in malaria in the Vihiga highland, nearly 1.3 times the overall rate in Kenya, largely due to the rise of drug-resistant strains of *P. falciparum* parasites [27, 28]. The ecology of the Vihiga highlands of Kenya supports stable transmission (thus is holoendemic) and increasing population pressure has led to agricultural changes creating ideal conditions for malaria vector proliferation [29]. Finally, *Anopheles* mosquitoes are generally highly zoophilic, rather than anthropophilic, thus becoming efficient human malaria vectors in Vihiga highland [30].

### Sample size determination

The sample size was determined using the formula *n =* Z^2^pq/d^2^ [31].

Where:

*n =* the sample size required,

z = 1.96: confidence level test statistic at the desired level of significance,

*p* = 52.0%: prevalence of malaria [28].

q = 1-p: proportion of malaria uninfected and d = 0.05: acceptable error willing to be committed.

d = acceptable standard error of the mean.

Therefore *n =* (1.96 × 1.96 × 0.52 × 0.48) ÷ 0.05^2^.

The optimum sample size estimated was *n =* 384. Therefore, this study required at least 384 study participants.

### Study participants and sample collection

Children were recruited via random sampling method and stratified into either severe malaria anaemia (SMA) and non-severe malaria anaemia (non-SMA) [32] or uninfected healthy controls. Socio-demographic information such as age, sex and anti-malarial therapy were collected using a questionnaire (See Supplementary I). *Plasmodium falciparum* malaria positive children who had received anti-malarial treatment within 48 h prior to the microscopical confirmation of their blood slides for malaria parasites and children co-infected with *P. falciparum* and other species of plasmodium, and Human Immunodeficiency virus type 1 (HIV-1), Hepatitis B virus (HBV) and Hepatitis C virus (HCV) were excluded from the study. Approximately, 2.0 mL of blood was collected in anticoagulant tube from each study participant and used for HIV-1/2, HBV and HCV serological testing, [33] haemoglobin measurement, and microscopy malaria diagnosis. Haemoglobin measurements were determined using Hb Hemocue 301 (Kuvettgattan 1,SE-26271 Angelholm Sweden) within 10 min from the time of blood collection to minimize variability in the measurements. The system was calibrated every morning before sample analysis.

### Malaria diagnosis

Thick and thin blood films were prepared from venous blood, stained with 10% Giemsa stain for 10 min and examined under a microscope. Parasite densities were calculated using the thick films by the WHO method (parasite count × 8000 divided by the number of WBCs counted which was 200) [32], and the thin films were used to establish the species of the parasites present. Films were classified as negative when no parasites are seen after two hundred microscopic fields had been examined. For purposes of quality control, slides were cross-examined independently by a senior microscopist and the results compared.

### ABO blood group determination

ABO blood groups typing was performed by forward grouping using commercial antisera (Biotech laboratories Ltd., Ipswich, Suffolk, UK) according to manufacturer’s protocol. Briefly, one drop of whole blood was placed in three different places on a grease-free clean glass slide labelled A, B and D. A drop of antisera A was added on the area labelled A, anti-B on B and anti-D on D. The blood cells and the antisera were mixed using a wooden applicator stick. The slide was then tilted to check for agglutination and result recorded accordingly.

### Hemoglobin genotyping

Haemoglobin genotypes were determined by cellulose acetate electrophoresis with Titan III plates according to the manufacturer’s protocols (Helena Bio-Sciences, Oxford, United Kingdom). Haemolysates prepared from blood samples and Hemo AFSC controls was dispensed onto the acetate paper, and haemoglobin variants separated by electrophoresis with an alkaline buffer at pH 8.6. The plates were then stained using Ponceau S stain, and haemoglobin genotypes scored using the Hemo AFSC control.

### G6PD genotyping

Glucose-6-phosphate dehydrogenase (G6PD) deficiency was determined by a fluorescent spot test (Trinity Biotech Plc., Bray, Ireland) as per the manufacturer’s protocol. Blood was hemolyzed and spotted onto a filter paper. Assay solution containing glucose-6-phosphate and oxidized Nicotinamide adenine dinucleotide phosphate (NADP^+^) was added, and samples excited with ultraviolet (UV) light at 340 nm. Based on the presence or absence of fluorescence emissions, the samples were scored as normal (high emission), intermediate (moderate emission), or deficient (no emission).

### Statistical analysis

Statistical analysis was performed using SPSS version 19.0 (IBM Corp., NY, USA). Proportions of sex between groups were determined by Chi-square test. Age, haemoglobin, parasitaemia, white blood cells (WBC), glucose and red blood cells (RBCs) levels between groups were determined using Mann-Whitney U test. Odds ratios were calculated with 95% confidence interval (CI) using logistic regression analyses. Statistical significance was set at *P* ≤ 0.05.

## Results

### Demographic and laboratory parameters

The demographic and laboratory measurements of the study participants are presented in Table 1. A total of 574 children were enrolled into this study comprising of 137 with Severe Malaria Anaemia (SMA, Hb < 5.0 g/dL with any parasite density) and 437 non-severe malaria anaemia (Hb ≥ 5.0 g/dL with any density parasitaemia). Sex distribution was not significant across the study groups (*P* = 0.540). Those with the SMA, 11.3 (11.6) mos. Were comparatively younger than those with non-SMA 14.6 (10.5) mos., *P* = 0.004. Haemoglobin concentrations were lower in children with SMA 4.2 (1.3) relative to the non-SMA 8.5 (2.2), *P* < 0.001. The concentrations of the white blood cells were higher in the SMA group; 13.5 (8.8) relative the non-SMA group 12.1 (6.2), *P <* 0.001. Furthermore, RBC counts were lower in children with SMA; 2.4 (0.8) when compared to the non-SMA group 3.5 (0.7), *P* < 0.001. Glucose concentrations were however similar between the two groups, *P* = 0.584.

**Table 1:** Demographic and clinical characteristics of study participants

Characteristics	SMA (Hb < 5.0 g/dL) (*n =* 137)	Non-SMA (Hb ≥ 5.0 g/dL) (*n* = 437)	*P-*value
**Demographic Characteristics**			
Sex			
Females	68 49.6)	230 (52.6)	0.540^a^
Males	69 (50.4)	207 (47.4)	
Age in months	11.3 (11.6)	14.6 (10.5)	**0.004**^**b**^
**Laboratory information**			
Hb, g/dL	4.2 (1.3)	8.5 (2.2)	**< 0.001**^**b**^
Parasite (/μL)	30,750.0 (102,680.5)	42,312.0 (125,216.8)	< 0.093 ^b^
WBCx10^3^/uL	13.5 (8.8)	12.1 (6.2)	**< 0.001**^**b**^
Glucose, mmol/L	6.0 (1.5)	5.8 (1.9)	0.584 ^**b**^
RBCx10^12^/μL	2.4 (0.8)	3.5 (0.7)	**< 0.001**^**b**^

Data are presented as medians (IQR, interquartile range) or indicated as number (n) and proportion (%) of subjects. ^**a**^Pearson’s Chi-square test for sex distribution. ^**b**^Statistical analysis was performed using Mann-Whitney U test

### Influence of ABO blood group on *P. falciparum* malaria in children under 3 years in Vihiga County, Kenya

The prevalence of the ABO blood groups in the study population were as follows O; 254/574 (42.7%), B; 152/574 (26.4%), AB; 22/574 (3.8%) and A; 156/574 (27.1%). We then determined the distribution of the various blood groups within the study groups. Results demonstrated no significant differences between the blood groups within the study stratifications (*P* = 0.331). To further determine the influence of ABO blood group on *P. falciparum* malaria in children, we performed a multinomial logistics regression using the common O blood group as the reference group. At 95%CI, the B, AB and A blood groups did not significantly influence outcome of malaria i.e. B; (OR = 0.811, 95%CI = 0.507–1.296, *P* = 0.380), AB; (OR = 2.750, 95%CI = 0.621–12.173, *P* = 0.183) and A; (OR = 0.839, 95%CI = 0.526–1.341, *P* = 0.464), respectively, Table 2.

**Table 2:** Influence of ABO blood group on *P. falciparum* malaria in children under 3 years in Vihiga County, Kenya

Category	Prevalence (%)	SMA (*n =* 137)	Non-SMA (*n =* 437)	*P- Value*^*a*^	OR	95% CI	*P-Value*^***b***^
**Blood groups**					**SMA**		
O	254 (42.7)	55 (9.6)	190 (33.1)	0.331	*Ref*	–	–
B	152 (26.4)	40 (7.0)	112 (19.5)		0.811	0.507–1.296	0.380
AB	22 (3.8)	2 (0.3)	19 (3.3)		2.750	0.621–12.173	0.183
A	156 (27.1)	40 (7.0)	116 (20.2)		0.839	0.526–1.341	0.464

Prevalence in the blood group genotypes in population studied are presented as n (percentage, %). Data shown are number (n) and proportions (%) of study participants. Odds ratios (OR) and 95% confidence intervals (95% CI) were determined using multivariate logistic regression controlling for age and sex. SMA, severe malaria anaemia. A, blood group A. B, blood group B. AB, blood group AB. O, blood group O

### Influence of glucose-6-phosphate dehydrogenase on *P. falciparum* malaria infection in children under 3 years in Vihiga County, Kenya

Before determining the influence of the G6PD on malaria infection, we performed prevalence analysis. The data show that normal G6PD genotype were 360/574 (62.7%), intermediate genotype 201 (35.0%) and deficiency in G6PD was 13/574 (2.3%). Glucose-6-phosphate deficiency showed a significant differences between the two study groups. The carriage of intermediate status of the G6PD was higher in the SMA group relative to the non-SMA. Moreover, there were more individuals who were deficient of G6PD in the non-SMA group compared to SM (*P* = 0.040). Further, regression analysis revealed that the carriage of the intermediate status of G6PD was associated with risk to SMA (OR = 1.52, 95%CI = 1.029–2.266, *P* = 0.035). However, the carriage of the G6PD deficient status was not associated with SMA (OR = 0.306, 95%CI = 0.039–2.392, *P* = 0.259), Table 3.

**Table 3:** Influence of glucose-6-phosphate dehydrogenase on *P. falciparum* malaria infection in children under 3 years in Vihiga County, Kenya

Category	Prevalence (%)	SMA *n =* 137	Non-SMA *n =* 437	*P*-Value^a^	OR	95% CI	*P*-Value^b^
**G6PD deficiency**					**SMA**		
Normal	360 (62.7)	77 (56.2)	283 (64.8)	**0.040**	*Ref*	–	–
Intermediate	201 (35.0)	59 (43.1)	142 (32.5)		1.527	1.029–2.266	**0.035**
Deficient	13 (2.3)	1 (0.7)	12 (2.7)		0.306	0.039–2.392	0.259

Prevalence in the G6PD genotypes in population studied are presented as n (percentage, %). Data shown are number (n) and proportions (%) of subjects. Odds ratios (OR) and 95% confidence intervals (95% CI) were determined using multivariate logistic regression controlling for age and sex. Non-severe malaria anaemia was used as the reference category. G6PD, glucose-6-phosphate dehydrogenase. Normal, normal G6PD. Intermediate, heterozygous G6PD. Deficient, homozygous G6PD. SMA, severe malaria aneamia; Non-SMA, non-severe malaria anaemia

### Influence of haemoglobin genotype on *P. falciparum* infection in children under 3 years in Vihiga County, Kenya

The prevalence of haemoglobin genotypes were as follows; AA 477/574 (83.1%), AS 93/574 (16.2%) and SS 4/574 (0.7%), respectively. Assessment of carriage of the sickle cell genetic variations revealed that there were 123 (89.8%) in SMA group and 354 (81%) of the AA in non-SMA group. Of the AS variation, there were 11 (8.0%) in the SMA group while 82 (18.8%) were in the non-SMA group (Table 4). Importantly, the variant SS were 3 (2.2%) in SMA group and 1 (0.2%) in non-SMA group. These differences were statistically significant (*P* = 0.001). When multinomial logistics regression was done with the AA as the reference group, the AS showed marginal association with protection against SMA (OR = 0.116, 95% CI = 0.012–1.124, *P* = 0.063). The carriage of the SS group could not run in the model to give a significant statistical meaning (Table 4).

**Table 4:** Influence of haemoglobin genotype on *P. falciparum* infection in children under 3 years in Vihiga County, Kenya

Category	Prevalence	SMA *n* = 137	Non-SMA *n* = 437	*P- Value*^*a*^	OR	95% CI	*P-Value*^*b*^
**Haemoglobin Status**					SMA		
AA	477 (83.1)	123 (89.8)	354 (81.0)	**0.001**	*Ref*	–	–
AS	93 (16.2)	11 (8.0)	82 (18.8)		0.116	0.012–1.124	0.063
SS	4 (0.7)	3 (2.2)	1 (0.2)		XX	XX	**XX**

Prevalence in the haemoglobin genotypes in population studied are presented as n (percentage, %). Data shown are number (n) and proportions (%) of subjects. Odds ratios (OR) and 95% confidence intervals (95% CI) were determined using multivariate logistic regression controlling for age and sex. Non-severe malaria anaemia was used as reference category. SMA, severe malaria, non-severe malaria anaemia. AA, normal hemoglobin. AS, heterozygote sickle cell. SS, homozygote sickle cell. **XX**, multivariate logistic regression was not performed because the numbers were too few to run in the model

### Influence of co-inheritance of haemoglobin and G6PD genotypes on *P. falciparum* infection in children under 3 years in Vihiga County, Kenya

The distributions of the coinheritance of the Hb and G6PD variations are as presented in Table 5. Multinomial regression analysis of the co-inheritance of both the sickle cell trait and the G6PD variations revealed that the AA/Intermediate with reference to AA/normal G6PD had increased risk to SMA (OR = 1.536, 95%CI = 1.007–2.343, *P* = 0.046). In addition, the AS/normal showed protection against SMA (OR = 0.337, 95%CI = 0.156–0.915, *P =* 0.031). These findings show important influence of co-inheritance in malaria disease outcome.

**Table 5:** Influence of co-inheritance of haemoglobin and G6PD genotypes on *P. falciparum* infection in children under 3 years in Vihiga County, Kenya

Category	SMA (*n* = 137)	Non-SMA (*n* = 437)	*P- Value*^*a*^	OR	95% CI	*P-Value*^*b*^
**Co-inheritance of Haemoglobin and G6PD deficiency**				SMA		
AA/Normal	69 (50.4)	230 (52.6)	**0.003**	*Ref*	–	–
AA/Deficient	1 (0.7)	9 (2.1)		0.370	0.046–2.975	0.350
AA/Intermediate	53 (38.7)	115 (26.3)		1.536	1.007–2.343	**0.046**
AS/Deficient	0 (0.0)	3 (0.7)		xx	xx	xx
AS/Intermediate	5 (3.6)	26 (5.9)		0.641	0.237–1.732	0.381
AS/Normal	6 (4.4)	53 (12.1)		0.377	0.156–0.915	**0.031**
SS/Intermediate	1 (0.7)	1 (0.7)		XX	XX	XX
SS/Normal	2 (1.5)	0 (0.0)		XX	XX	XX

Data shown are number (n) and proportions (%) of subjects. ^a^Statistical comparison was performed using Pearson’s Chi-square test. Odds ratios (OR) and 95% confidence intervals (95% CI) were determined using multivariate logistic regression controlling for age and sex. SMA, severe malaria anaemia, non-severe malaria anaemia. AA, normal hemoglobin. AS, heterozygote sickle cell. SS, homozygote sickle cell. **XX**, multivariate logistic regression was not performed because the numbers were too few to run in the model

## Discussion

Malaria remains one of the most important causes of morbidity and mortality in children leaving in endemic areas [1]. Highest mortality occurs in young children with under-developed protective immune mechanisms against the parasite. It has been previously shown that severe malaria anaemia accounts for 12% of all malaria related deaths [34]. A minority of children with certain red blood cell variants have a natural biological advantage thought to partially hamper parasite growth [21]. Therefore, it was imperative to investigate the effect of ABO, G6PD and haemoglobin variants on highland malaria in Kenyan children resident in western Kenya.

In the present study, we did not find a significant association between carriage of the blood group genotypes and severe malaria outcome in children. This finding is, however, inconsistent with previous studies involving Ghanaian children [9] in which blood group O showed partial protection. It has been shown that blood group O protects against malaria through reduced resetting [35] and by inducing high levels of anti-malarial IgG antibodies which directly inhibit parasite invasion or growth in erythrocytes, or indirectly by a mechanism involving cooperation between parasite-opsonising antibody and monocyte [36, 37]. Certain studies have reported absence of association between the ABO blood group system and *P. falciparum* malaria infection among children in Nigeria [8]. Other previous in vitro erythrocyte preference assays demonstrated that *P. falciparum* parasites prefer type O over type A erythrocytes [9]. These findings countered the known protective effect of group O against severe malaria, but additionally emphasised the complexities of host-pathogen interactions, and the need for highly quantitative and scalable assays. However, recent meta-analyses data involving 1923 articles obtained from the databases, demonstrated that ABO blood group may not affect susceptibility to asymptomatic and/or uncomplicated *P. falciparum* infection but showed that blood group O primiparous women appeared to be more susceptible to active placental *P. falciparum* infection [38]. Additional meta-analyses demonstrated that the difference in the level of *P. falciparum* parasitaemia was not significant among individuals with blood group A or non-O compared with blood group O. Furthermore, the difference in haemoglobin level among *P. falciparum* infected individuals was also not significant between those with blood group A, B or AB versus those with blood group O [39]. These inconsistencies maybe be due to geographic and ethnic distribution of the different ABO genetic blood polymorphisms associated with malaria protection in the regions [25].

Our current study did not show a significant association between the haemoglobin genotypes and malaria disease outcome. The findings of this study are consistent with previous studies reporting no association between haemoglobin AA, AS and SS genotype with malaria infection among children in Nyando County, Kenya [14]. However, these findings are inconsistent with other previous findings in Kenyan coast and Siaya which showed that children with haemoglobin AA have increased risk of developing severe malarial disease relative to children with haemoglobin SS in Kenya [13, 40–42]. These differences may be attributed to the extreme variability of α-globin and β-globin malaria resistance genes attributed to high amounts of standing variation or high mutation rates rapidly producing new adaptive alleles [43]. However, this phenomenon was not investigated in the current study.

The findings of this study showed that intermediate status of G6PD was associated with SMA. In addition the carriage of other G6PD variations did not show any association with malaria disease outcome. It is also important to note that genetic impact on infectious diseases is multigenic and not a single gene might reveal the actual effect. This is partly consistent with previous studies in Kilifi, Kenya, and Tanzania which showed protection with this particular variant in females but not males [18, 25]. However, other studies in Mali involving children with severe malaria indicated that hemizygous males and possibly, homozygous but not heterozygous females are protected from malaria [20] suggesting that the distribution of G6PD polymorphism is influenced by geographic area, sex and ethnic group and that genetic impact on infectious diseases is multigenic and not a single gene might reveal the actual effect.

We show in the current study that the co-inheritance of haemoglobin AA variations and G6PD intermediate status is associated risk of severe malaria while the carriage of the sickle cell trait and normal G6PD is associated with protection against severe malaria. This reveal that the effect of one gene on the other and that genes act in combination. This is an important finding that its mechanisms are worth investigation.

It is important to outline the limitations of our study. Several red blood cell polymorphisms, including those linked to pyruvate kinase, complement receptor-1 and haemoglobinopathies such as thalassaemia traits, have a role in the clinical outcome of malaria [42] and might be present among the population. Even though a prospective study would have been important in determining the effect of inherited blood disorders on malaria, this cross-sectional study demonstrated association between ABO, G6PD and haemoglobin type on malaria in Kenyan children.

## Conclusion

This study reveals that blood groups do not have a significant influence on malaria disease outcome. Haemoglobin AS offers rather a marginal protection against severe compared to the normal haemoglobin. Carriage of the heterozygous G6PD type compared to the normal G6PD is associated with risk of severe malaria anaemia. The co-inheritance of Hb variations and G6PD variation are important predictors of malaria disease outcome in this region. The findings of this study underline the potential of the Genome Wide Association approach to provide candidates for the development of control measures against malaria in humans.

## Supplementary information

**Additional file 1.**

## Data Availability

All data generated or analyzed during this study are included in this published article [and its supplementary information files].

## Abbreviations

ABO: ABO Blood group system
AFSC: Control for haemoglobin type A, F, S and C
CI: Confidence Interval
G6PD: Glucose-6-phosphate dehydrogenase
Hb: Haemoglobin
HBV: Hepatitis B Virus
HCV: Hepatitis C Virus
HIV-1: Human Immunodeficiency Virus type 1
OR: Odd Ratio
SMA: Severe Malarial anaemia
SPSS: Statistical Package for Social Scientists
RBC: Red Blood Cell
WBC: White Blood Cell
