# Supplementary material for: Influence of blood group, Glucose-6-phosphate dehydrogenase and Haemoglobin genotype on Falciparum malaria in children in Vihiga highland of Western Kenya
Source: BMC Infect Dis. 2020 Jul 9;20:487. doi: 10.1186/s12879-020-05216-y (PMC7346653; doi:10.1186/s12879-020-05216-y)
Supplement: Supplementary file 1 — Additional file 1. [file 12879_2020_5216_MOESM1_ESM.docx]

## Questionnaire

**Title: Influence of ABO blood group, glucose -6-phosphate dehydrogenase and haemoglobin genotype on *p. falciparum malaria infection* outcome in children under 3 years in Vihiga Kenya**

Study participant ID .......................................................................

Volunteer’s Name .......................................................................

Mother /Guardian ‘s Name .......................................................................

Date of Birth ........................................................Sex...........

Recruitment Date ...................................Age ..............................

**Contacts**

Telephone no. ............................. Village ......................................

County ................................... Nearest Market..........................

Location ................................... Nearest Church..........................

Chief ................................... Nearest School..........................

**Gender**…………………………….. **Ethnic group** ..............................................

Which of the following Malaria Prevention methods are used in the home(check all that apply)

Mosquito Nets [ ] Mosquito Repellant Gel [ ] None [ ]

Mosquito Coils/Sprays [ ] Other (specify) ...................................

**Presenting signs and symptoms/physical examination**

( yes /no)

Fever .............................. Convulsions...................................

Vomiting ............................ Unresponsiveness...........................

Poor Feeding ........................... Difficult breathing...........................

Headache ........................... Temperature...................................

Pallor ........................... Others..........................................

Jaundice ...........................

**Treatment history**

Has the child received any of the following medications in the past 48 hours?

(Yes /No)

Quinine .............................................................

Panadol .............................................................

Amodiaquine ...........................................................

Artemether/Lumefandrine........................... ...................

Others (specify) ....................................................

Was the child hospitalized.................................................

Has the child ever participated in this study before................

**Diagnosis/Clinical information**

Severe Malaria....................................... Healthy control..............................................

Uncomplicated Malaria.................................

Parasite density (if present)………………………………………………

RBCx10^12^/µL………………………….

WBCx10^3^/uL…………………………..

Glucose level (mmol/L)  ……………....

Hb level (g/dL)…………………………
